# Prognostic Significance of Systemic Immune-Inflammation Index in Patients With Diffuse Large B-Cell Lymphoma

**DOI:** 10.3389/fonc.2021.655259

**Published:** 2021-05-26

**Authors:** Zanzan Wang, Jiawei Zhang, Shuna Luo, Xiaoying Zhao

**Affiliations:** ^1^ Department of Hematology, The Second Affiliated Hospital of Zhejiang University School of Medicine, Zhejiang University, Hangzhou, China; ^2^ Department of Hematology, The Fourth Affiliated Hospital of Zhejiang University School of Medicine, Zhejiang University, Yiwu, China

**Keywords:** diffuse large B-cell lymphoma, systemic immune-inflammation index, prognosis, neutrophil, lymphocyte, platelet, nomogram

## Abstract

**Objective:**

The systemic immune-inflammation index (SII) based on neutrophil, platelet and lymphocyte counts, is a prognostic biomarker in some solid cancers. However, the prognostic value of SII has not yet been validated. This study was to evaluate the role of SII in predicting survival for patients with diffuse large B cell lymphoma (DLBCL).

**Methods:**

We retrospectively investigated 224 patients with DLBCL between August 2005 and October 2018. Kaplan–Meier analysis and Cox proportional hazard models were used to assess the prognostic value of SII.

**Results:**

In the ROC curve analysis, SII had the highest AUC and was more accurate as a prognostic factor. Patients with higher SII tended to have higher level of LDH, more advanced stage, poor PS, and high IPI score compared with low SII group. In univariate analyses, SII, PLR and NLR were all prognostic for progression-free survival and overall survival. Moreover, only SII, older age, HBSAg-positive and IPI were the independent prognostic factors for patients in multivariate analysis. The nomogram based on SII, older age, HBSAg status and IPI showed accurate prognostic ability for predicting 3-years and 5-years survival rates (c-index, 0.791) compared to the IPI alone (c-index, 0.716).

**Conclusion:**

SII was a powerful tool for predicting outcome in patients with DLBCL. It might assist the separation of high-risk patients among patients with the same IPI.

## Introduction

Diffuse large B-cell lymphoma (DLBCL) is the most common histologic subtype of non-Hodgkin’s lymphoma (NHL), accounting for 30-40% of new cases. Over the recent years, due to the introduction of rituximab into treatment regimens, more than half of untreated DLBCL patients can be better cured (1). However, approximately 30%-40% patients still have recurrence or develop refractory disease that remain poor outcome (2). Therefore, it is significant to distinguish the patients with poor prognosis in the early stage and select the effective therapeutic regimen accordingly.

During the pre-rituximab era, International Prognostic Index (IPI) was the most powerful prognostic clinical tool for DLBCL (3). After the extensive use of rituximab, the outcome of DLBCL patients has been improved so much that it is difficult to identify high-risk groups using IPI alone (4). In an effort to risk-stratify patients treated with R-CHOP, the revised IPI (R-IPI) and NCCN-IPI were generated. The R-IPI redistribute the IPI clinical scores to form three groups, which has better prediction of clinical outcomes compared with IPI (5). The NCCN-IPI is also calculated based on various clinical characteristics with better definition of extranodal site involvement and re-evaluation of age and LDH to divide patients into four groups (6). Recently, molecular genetic markers (7) and gene expression profiling (8) have been identified as new prognostic parameters. However, these indicators are inconvenient and expensive, so it is essential to seek readily available and inexpensive parameters to stratify the prognosis of patients with DLBCL and provide appropriate treatment measures.

Inflammation plays a vital role in the tumor progression and therapeutic response. Peripheral blood counts which in some degree reflect inflammation status are closely related to the progress of cancers (9, 10). Numerous studies published in recent years have demonstrated that inflammation indicators such as pretreatment neutrophil-lymphocyte ratio (NLR) (11) and platelet-lymphocyte ratio (PLR) (12) play crucial roles in influencing the outcomes of the DLBCL patients. Moreover, compared with molecular genetic markers, blood biomarkers are cheaper and easier to obtain.

High systemic immune-inflammation index (SII), which is associated with neutrophil, platelet and lymphocyte counts, has been reported to be a poor prognostic indicator in several solid malignancies, such as pancreatic cancer (13), breast cancer (14), lung cancer (15) and gastrointestinal cancer (16). But the cutoff of SII in different cancers is different. To the best of our knowledge, the relationship between SII and the outcome of DLBCL has not been explored. Hence, we first conducted this study to evaluate the clinical and prognostic value of SII in patients with DLBCL.

## Materials and Methods

### Patients

We retrospectively reviewed the data of 436 patients with DLBCL at the Second Affiliated Hospital of Zhejiang University School of Medicine between August 2001 and October 2018. 55 patients with incomplete data and 157 patients received CHOP or CHOP-like chemotherapy were excluded. Included in the study were 224 patients with DLBCL between August 2005 and October 2018. A total of 124 DLBCL patients from 2005 to 2018 from the Second Affiliated Hospital of Zhejiang University School of Medicine were reviewed as the validation cohort. We included 90 patients with complete data, while 22 patients received CHOP or CHOP-like chemotherapy and 12 patients with incomplete data were excluded. Selection criteria were as follows (1): patients were confirmed CD20+ DLBCL according to the 2016 WHO Classification of Tumours of Haematopoietic and Lymphoid Tissue (17) (2); patients who received at least 4 cycles of R-CHOP (rituximab, cyclophosphamide, doxorubicin, vincristine, and prednisone),or R-CEOP (rituximab, etoposide, cyclophosphamide, vincristine, and prednisone) and R-EPOCH (rituximab, etoposide, cyclophosphamide, doxorubicin, vincristine, and prednisone) (3). the availability of complete information in laboratory tests and medical records. All patients who were suspected of being infected must receive examinations including CRP, procalcitonin, CT of the infected site, etc. to determine whether they were infected. Patients with infection at initial diagnosis were excluded. Patients were excluded if they were HIV positive. Transformed indolent lymphoma and primary central nervous systems (CNS) B cell lymphoma were excluded. Primary mediastinal large B-cell lymphoma, high grade cell lymphoma and intravascular large B-cell lymphoma were also excluded. This study was approved by the Ethics Committee of the Second Affiliated Hospital of Zhejiang University School of Medicine in line with the principles of the Declaration of Helsinki.

### Data Collection

We retrieved patient characteristics including gender, age, LDH level, Ann Arbor stage, B symptoms, serum β_2_-microglobulin, IPI, Eastern Cooperative Oncology Group Performance Status (ECOG PS), bulky disease(≥7.5cm), hepatitis B surface antigen status, number of extranodal involvement and chemotherapy regimen. The laboratory data and full blood cell counts were obtained before 1 week of the treatment of DLBCL. SII was defined as platelet counts × neutrophil counts/lymphocyte counts. PLR and NLR were calculated by dividing the absolute platelet counts and neutrophil counts by the absolute lymphocyte counts, respectively. Follow-up of all patients ended on December 20, 2020. During the follow-up time, 38 patients in the group were lost to follow-up in the entire cohort. The missing rate was 17.0%. These patients who lost to follow-up were censored on their own last follow-up visit. Overall survival (OS) was calculated as the interval between diagnosis and death or date of last follow-up and progression free survival (PFS) was calculated from diagnosis to recurrence, progression, death from any cause or last follow-up.

### Statistical Analyses

ROC curves were used to determine the optimal cut-off values and measure the area under curve (AUC) of NLR, SII and PLR. The categorical variables were analyzed by the Pearson Chi-squared test. The association between the different groups with OS and PFS was analyzed using the Kaplan–Meier curves and compared using the log-rank test. Univariate and multivariate analysis were used to evaluate prognostic values of each variable. The Cox proportional hazards regression model was used to examine the hazard ratio (HR) and 95% confidence interval (95% CI). Predictors with p < 0.05 were considered statistically significant, which were incorporated into the nomogram. The concordance index (c index) was calculated to evaluate the ability of the nomogram. Next, model calibration was used to verify the accuracy of nomogram by comparing the predicted OS with the actual OS. SPSS statistical software (SPSS statistics 25.0), R software (version 3.6.3) and GraphPad Prism software were used for statistical analyses.

## Results

### Patient Cohorts and Characteristics

The clinical characteristics of 224 patients with confirmed DLBCL included in this study were showed in **Table 1**. The median age was 59 years (range 22 – 80 years), with 49.1% of patients older than 60 years of age. Female/male ratio was 0.85:1. B symptoms was observed in 65 (29.0%) patients. 148 (66.1%) patients had more advanced stage, and 54 (24.1%) patients had performance status ECOG ≥2. The patients for low, low-intermediate, high-intermediate, high IPI scores were 70 (31.3%), 63 (28.1%), 46 (20.5%) and 45 (20.1%), respectively. R-CHOP was the most commonly used regimen (n=199, 88.9%), followed by R-CEOP (n=24, 10.7%), R-EPOCH (n=1, 0.4%). The median values of absolute neutrophil counts, absolute lymphocyte counts, absolute platelet counts were 3.795(1.08~21.07)×10^9^/L, 1.295(0.2~6.8)×10^9^/L and 210 (53~456)×10^9^/L. Most patients (192, 85.7%) obtained complete remission (CR) or partial response (PR), while relapse after response occurred in 48 (25.0%) patients. During the median follow-up time of 51.3 months, 57 (25.4%) died before the end of the follow-up period.

**Table 1:** Baseline characteristics of the study population categorized by systemic immune-inflammation index(SII).

Characteristics	Overall N (%)	SII ≥1046.1N (%)	SII＜1046.1N (%)	p
N	224	55	169	
Age (years)				
<60	114 (50.9)	29 (52.7)	85 (50.3)	0.754
≥60	110 (49.1)	26 (47.3)	84 (49.7)	
Gender				
Male	121 (54.0)	32 (58.2)	89 (52.7)	0.476
Female	103 (46.0)	23 (41.8)	80 (47.3)	
LDH				
Increased	121 (54.0)	47 (85.5)	74 (43.8)	**<0.001**
Normal	103 (46.0)	8 (14.5)	95 (56.2)	
B-symptoms				
No	159 (71.0)	34 (61.8)	125 (74.0)	0.085
Yes	65 (29.0)	21 (38.2)	44 (26.0)	
Ann Arbor stage				
I/II	76 (33.9)	11 (20.0)	65 (38.5)	**0.012**
III/IV	148 (66.1)	44 (80.0)	104 (61.5)	
Performance status				
0-1	170 (75.9)	30 (54.5)	140 (82.8)	**<0.001**
≥2	54 (24.1)	25 (45.5)	29 (17.2)	
Bulky disease				
0	215 (96.0)	52 (94.5)	163 (96.4)	0.532
1	9 (4)	3 (5.5)	6 (3.6)	
Extranodal involvement				
<1	45 (20.1)	9 (16.4)	36 (21.3)	0.427
≥1	179 (79.9)	46 (83.6)	133 (78.7)	
HBSAg				
Negative	181 (80.8)	44 (80.0)	137 (81.1)	0.862
positive	43 (19.2)	11 (20.0)	32 (18.9)	
IPI				
Low	70 (31.3)	6 (10.9)	64 (37.8)	**<0.001**
Low-intermediate	63 (28.1)	14 (25.5)	49 (29.0)	
High-intermediate	46 (20.5)	17 (30.9)	29 (17.2)	
High	45 (20.1)	18 (32.7)	27 (16.0)	
NLR				
<3.554	142 (63.4)	5 (9.1)	137 (81.1)	**<0.001**
≥3.554	82 (36.7)	50 (90.9)	32 (18.9)	
PLR				
<216.00	162(72.3)	5(9.1)	157(92.9)	**<0.001**
≥216.00	62(27.7)	50(90.9)	12(7.1)	
Response to treatment				
CR+PR	192 (85.7)	42 (76.4)	150 (88.8)	**0.023**
SD+PD	32 (14.3)	13 (23.6)	19 (11.2)	

GC, germinal center; LDH, lactate dehydrogenase; IPI, International Prognostic Index; CR, complete remission; PR, partial response; SD, stable disease; PD, progressive disease. Significant p value are represented in bold.

**Figure 1:**
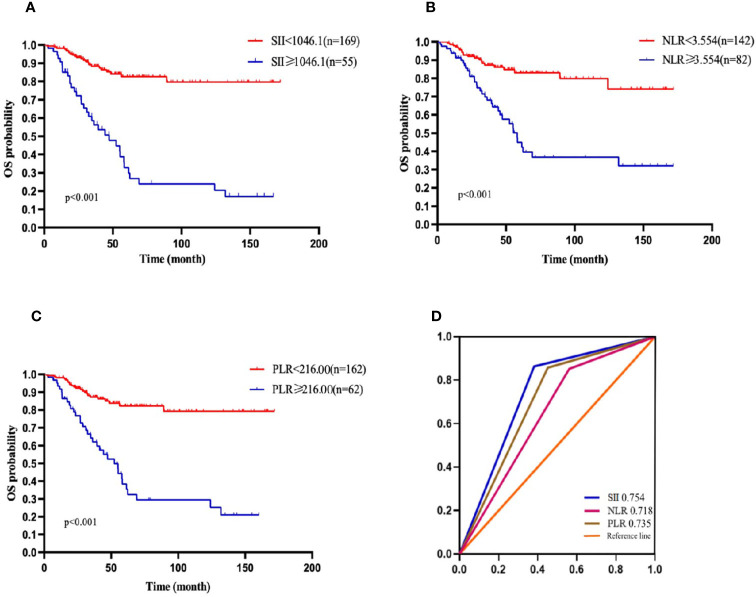
OS of patients with diffuse large B cell lymphoma (DLBCL). **(A)** OS in patients affected by DLBCL with SII at diagnosis < 1046.1 and ≥ 1046.1. **(B)** OS in patients affected by DLBCL with NLR at diagnosis < 3.554 and ≥ 3.554. **(C)** OS in patients affected by DLBCL with PLR <216.00 and ≥ 216.00 at diagnosis. **(D)** Predictive ability of the SII in DLBCL was compared with PLR and NLR by ROC curves in 3-years in the primary cohort.

According to the ROC curve, the optional cutoff points of SII, NLR and PLR were 1046.1, 3.554 and 216.00 (**Figure 1D**). The area under the ROC curves for SII, NLR and PLR were 0.754, 0.718 and 0.735, respectively, demonstrating that the prognostic value of SII was better than that of PLR and NLR.

Patients were divided into two groups according to the cutoff value: 169 patients had SII <1046.1 and 55 patients had SII ≥ 1046.1. Patients with high SII tended to have high level of LDH, more advanced stage, poor PS, and high IPI score. Moreover, 42 (76.4%) patients with high SII achieved CR or PR and 150 (88.8%) patients with low SII achieved CR or PR, which showed the patients with high SII had statistically significant poor response (p<0.001). However, there were no significant differences between two group in sex, age, B-symptoms, bulky disease, extranodal involvement and HBSAg status.

### Univariate and Multivariate Analysis of OS

In the present study, the 1-, 2-,3-, and 5-year OS rates were 96.4%, 88.2%, 81.1%, and 68.8%, respectively. Patients with SII <1046.1 (n=169) had a 3-year OS probability of 88.3%, significantly higher than the 3-year OS rate of 58.6% in patients with SII ≥ 1046.1 (n=55) (p=0.001) (**Figure 1A**). The survival curves of PLR and NLR also showed similar trends(**Figures 1B, C**). Furthermore, the univariate analysis revealed that older age, male, high level of LDH, high IPI score, ECOG≥2, HBsAg- positive, high NLR, high PLR and high SII had significantly inferior OS.

Multivariate Cox proportional hazard models were used to analyze whether the above parameters were effective predictors. In multivariate analyses, older age, HBsAg -positive, high IPI score and high SII were independent prognostic factors for poor OS (**Table 2**).

**Table 2:** Univariate and multivariate analysis of prognostic factors for OS in DLBCL patients.

Variable	Parameter	Univariate analysis		Multivariate analysis	
		HR (95% CI)	p value	HR (95% CI)	p value
Age	<60	1	**0.018**	1	**0.023**
	≥60	1.864 (1.105-3.145)		2.109 (1.109-4.010)	
Gender	Female	1	**0.035**	1	0.351
	Male	1.828 (1.086 -3.078)		1.327 (0.732-2.407)	
B symptoms	Absent	1	0.084		
	Present	1.607(0.883-2.922)			
Performance status	0-1	1	**<0.001**	1	0.967
	≥2	3.002 (1.536-5.867)		1.014(0.533-1.928)	
LDH	Normal	1	**0.003**	1	0.073
	Increased	2.299 (1.367-3.864)		1.963(0.940-4.102)	
IPI	Low	1		1	
	Low-intermediate	4.525 (1.606-12.747)	**0.004**	3.583 (1.209-10.614)	**0.021**
	High-intermediate	11.293(4.131-30.873)	**<0.001**	7.091(2.307-21.978)	**0.001**
	High	11.352(4.114-31.328)	**<0.001**	6.620(1.863-23.515)	**0.003**
Bone marrow involvement	No	1	0.100		
	Yes	2.002 (0.644-6.223)			
Extranodal involvement	<1	1	0.689		
	≥1	1.143 (0.678-2.152)			
Ann Arbor stage	I/II	1	0.085		
	III/IV	1.711 (0.988-2.965)			
HBsAg	Negative	1	**0.005**	1	**0.002**
	Positive	2.167 (1.095-4.289)		2.656 (1.414.976)	
SII	<1046.1	1	**<0.001**	1	**0.029**
	≥1046.1	5.659(2.982-10.740)		3.850 (3.850-12.941)	
NLR	<3.554	1	**<0.001**	1	0.534
	≥3.554	3.608(2.070-6.289)		1.299(0.570-2.960)	
PLR	<216.00	1	**<0.001**	1	0.919
	≥216.00	4.686(2.551-8.607)		1.058(0.356-3.141)	

IPI International Prognostic Index; LDH lactic dehydrogenase; SII systemic immune-inflammation index; HR hazard ratio; CI confidence interval. The bold values indicate that p < 0.05 and the corresponding factors are significantly associated with survival.

### Univariate and Multivariate Analysis of PFS

**Figure 2:**
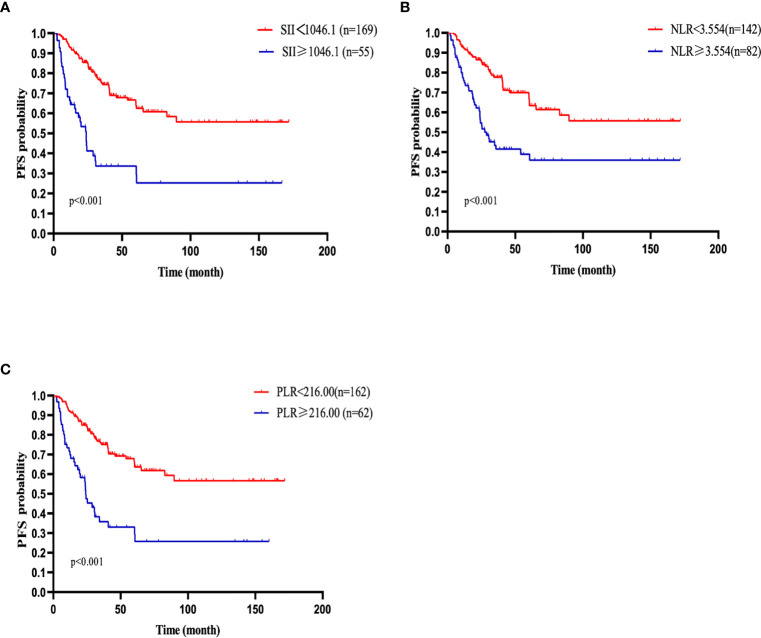
PFS of patients with diffuse large B cell lymphoma (DLBCL). **(A)** PFS in patients affected by DLBCL with SII at diagnosis < 1046.1 and ≥ 1046.1. **(B)** PFS in patients affected by DLBCL with NLR at diagnosis < 3.554 and ≥ 3.554. **(C)** PFS in patients affected by DLBCL with PLR < 216.00 and ≥ 216.00 at diagnosis.

Similarly, the 1-, 2-, 3-, and 5-year PFS rates were 86.5%, 76.4%, 65.1%, and 59.1%, respectively. Patients with SII <1046.1 (n=169) had a 3-year PFS probability of 74.4%, higher than the 3-year PFS rate of 33.7% in patients with SII ≥ 1046.1 (n=55)(p<0.001). The survival curve also showed that the PFS of patients with high NLR and high PLR values were shorter than that of patients with low values (**Figures 2 A–C**). The univariate analysis demonstrated that older age, male, B symptoms, high level of LDH, ECOG≥2, high IPI score, advanced stage (stage III/IV), high NLR, high PLR and high SII were independent prognostic predictors of PFS. Multivariate Cox regression analysis indicated that high IPI score, high SII and high level of LDH were related to poor PFS (**Table 3**).

**Table 3:** Univariate and multivariate analysis of prognostic factors for PFS in DLBCL patients.

Variable	Parameter	Univariate analysis		Multivariate analysis	
		HR (95% CI)	p value	HR (95% CI)	p value
Age	<60	1	**0.003**	1	0.153
	≥60	1.961 (1.267-3.036)		1.434 (0.874-2.354)	
Gender	Female	1	**0.048**	1	0.205
	Male	1.553 (1.004 -2.402)		1.338 (0.853-2.101)	
B symptoms	Absent	1	**0.018**	1	0.507
	Present	1.701(1.096-2.640)		1.172 (0.733-1.875)	
Performance status	0-1	1	**<0.001**	1	0.627
	≥2	2.341 (1.492-3.674)		1.149(0.656-2.012)	
LDH	Normal	1	**0.019**	1	**0.023**
	Increased	1.294 (1.043-1.606)		1.926(1.094-3.392)	
IPI	Low	1		1	
	Low-intermediate	3.072 (1.536-6.143)	**0.002**	2.952 (1.330-6.551)	**0.008**
	High-intermediate	6.044(2.984-12.244)	**<0.001**	5.377(1.728-16.736)	**0.003**
	High	6.401(3.183-12.870)	**<0.001**	6.292(2.556-15.490)	**<0.001**
Bone marrow involvement	No	1	0.072		
	Yes	1.953 (0.942-4.052)			
Extranodal involvement	<1	1	0.924		
	≥1	1.026 (0.610-1.723)			
Ann Arbor stage	I/II	1	**0.019**	1	0.417
	III/IV	1.801 (1.102-2.943)		1.311 (0.682-2.520)	
HBsAg	Negative	1	0.101		
	Positive	1.509 (0.923-2.466)			
SII	<1046.1	1	**<0.001**	1	**0.043**
	≥1046.1	1.835(1.476-2.280)		2.253 (1.026-4.950)	
NLR	<3.554	1	**<0.001**	1	0.275
	≥3.554	1.609(1.302-1.987)		1.381(0.773-2.465)	
PLR	<216.00	1	**<0.001**	1	0.471
	≥216.00	1.785(1.441-2.212)		1.289(0.646-2.573)	

IPI International Prognostic Index; LDH lactic dehydrogenase; SII systemic immune-inflammation index; HR hazard ratio; CI confidence interval. The bold values indicate that p < 0.05 and the corresponding factors are significantly associated with survival.

### Nomogram Predicting Survival

**Figure 3:**
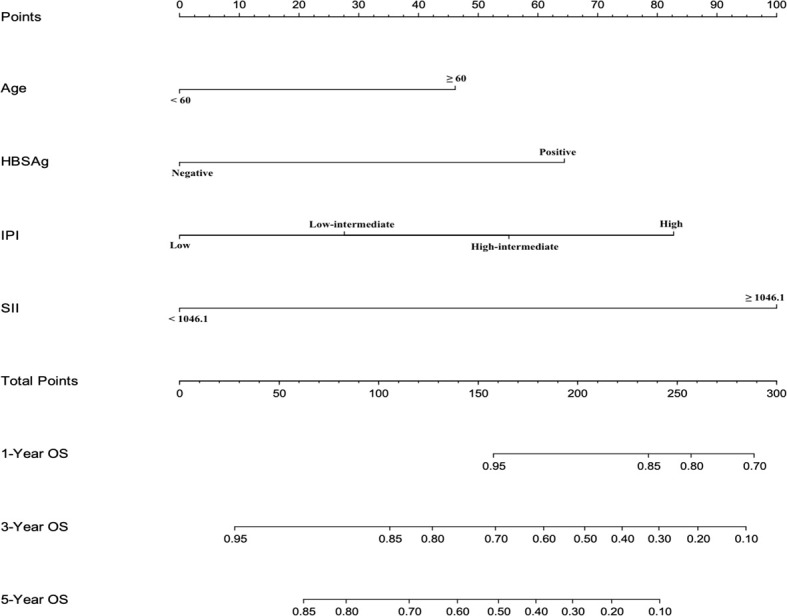
Evaluation of nomogram integrated age, SII, IPI and HBsAg status in patients with DLBCL.

**Figure 4:**
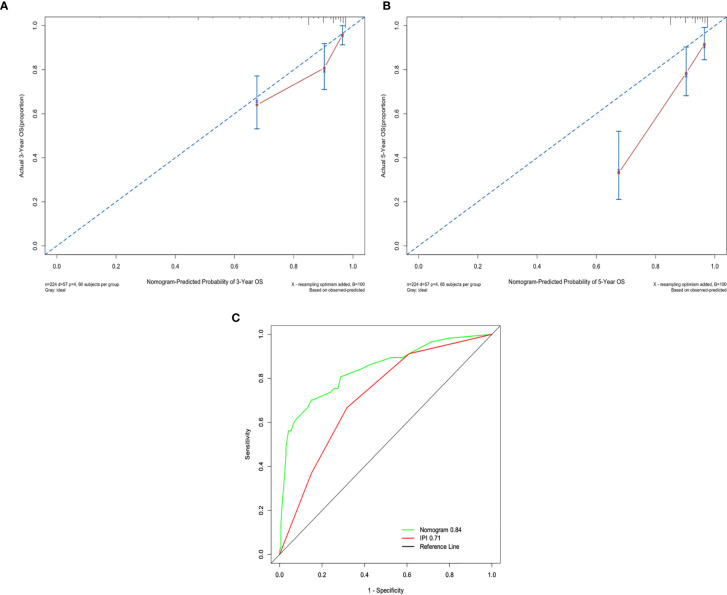
**(A)** The 3-years survival rate of DLBCL predicted by nomogram is consistent with the actual observed values. **(B)** The 5-years survival rate of DLBCL predicted by nomogram is consistent with the actual observed values. **(C)** The ability of ROC analysis nomogram to predict the 3-years survival rate of the DLBCL, the nomogram has a larger AUC than IPI.

A nomogram, including significant independent risk factors such as SII, IPI, age and HBSAg status, was established to predict the 3- and 5-years OS (**Figure 3**). The C-index of the nomogram was 0.791, which showed excellent role of predicting the outcome compared to the IPI (C-index, 0.716). The calibration curve of the nomogram showed great concordance between the predicted and actual survival rates for 3- and 5-years(**Figures 4A, B**). Moreover, according to the ROC curve, the AUC of the nomogram was 0.840, which was higher than that of IPI (AUC=0.718, p<0.001), illustrated that this nomogram was suited for predicting the outcome of DLBCL patients(**Figure 4C**).

### Validate the Prognostic Value of SII

We verified the role of SII in survival of the patients in the validation cohort. A total of 90 DLBCL patients were enrolled in this study as the validation cohort. The median age of the patients was 58 (22–80) years. All patients received R-CHOP regimen. Survival curve showed that patients with lower SII levels had better OS than those with higher levels of SII (p<0.001), which was consistent with the primary results. In univariate analysis, we found that older age, ECOG≥2, high level of LDH, HBsAg positive, high IPI scores, high NLR, high PLR and high SII were associated with poor OS. And in multivariate analyses, older age and high SII were shown to be independently associated with poor OS (**Table 4**). These results demonstrated that the SII could be used as an accurate prognostic factor for predicting the survival of patients.

**Table 4:** Univariate and multivariate analysis of prognostic factors for OS in DLBCL patients in validation cohort.

Variable	Parameter	Univariate analysis		Multivariate analysis	
		HR (95% CI)	p value	HR (95% CI)	p value
Age	<60	1	**<0.001**	1	**0.038**
	≥60	6.063 (2.287-16.071)		3.869 (1.076-13.917)	
Gender	Female	1	0.725		
	Male	1.146 (0.538-2.441)			
B symptoms	Absent	1	0.063		
	Present	2.075(0.962-4.478)			
Performance status	0-1	1	**0.034**	1	0.372
	≥2	2.286 (1.065-4.907)		1.535(0.599-3.934)	
LDH	Normal	1	**<0.001**	1	0.462
	Increased	4.494 (1.933-10.450)		1.501(0.509-4.427)	
IPI	Low	1		1	
	Low-intermediate	14.851 (1.713-128.715)	**0.014**	3.433(0.318-37.101)	0.310
	High-intermediate	34.050(4.238-273.577)	**0.001**	10.028(0.873-115.164)	0.064
	High	70.155(8.519-577.710)	**<0.001**	12.763(0.968-168.286)	0.053
Bone marrow involvement	No	1	0.141		
	Yes	2.224 (0.767-6.449)			
Extranodal involvement	<1	1	0.924		
	≥1	1.026 (0.610-1.723)			
Ann Arbor stage	I/II	1	0.084		
	III/IV	2.555 (0.883-7.395)			
HBsAg	Negative	1	**0.006**		
	Positive	3.344 (1.405-7.961)			
SII	<1046.1	1	**<0.001**	1	**0.002**
	≥1046.1	4.340(2.760-6.824)		79.091 (5.279-118.959)	
NLR	<3.554	1	**<0.001**	1	0.443
	≥3.554	2.259(1.493-3.418)		1.686(0.444-6.402)	
PLR	<216.00	1	**<0.001**	1	0.078
	≥216.00	2.148(1.433-3.218)		6.600(0.809-53.868)	

IPI International Prognostic Index; LDH lactic dehydrogenase; SII systemic immune-inflammation index; HR hazard ratio; CI confidence interval. The bold values indicate that p < 0.05 and the corresponding factors are significantly associated with survival.

## Discussion

Inflammation is closely linked to tumor development (18). Immune cells always secrete cytokine and chemokine to shape tumor growth in tumor microenvironment.

Neutrophils which exist in tumor microenvironment are capable of producing angiogenic chemokines and cytokines including CCL2,CCL3, CCL5, CCL10, IL4 and IL10 to promote tumor growth, invasion and angiogenesis (19). Therefore, high levels of neutrophils could affect the survival of DLBCL. In solid tumors, it has been found that platelets can protect circulating cancer cells from NK cell-mediated killing, promote cancer cells metastasis and secrete cytokines to stimulate proliferation of tumor cells (20, 21). Tumor-infiltrating lymphocytes could inhibit tumor cell proliferation and metastases, and low lymphocytes could weaken the immunological response to tumor (22). Lymphocytes are the basis of cytotoxic effect of rituximab on tumor cells (23). Previous studies have shown that a low lymphocyte count played an adverse impact on outcome in DLBCL patients (24). In the study by Kusano and colleagues, low CD4+ T-cell count at diagnosis was significantly related to OS and PFS, and the CD4+ T-cell count might be an independent predictive marker in patients with DLBCL (25). Dehghani et al. (26) also demonstrated that in patients with DLBCL receiving R-CHOP, the elevated level of Treg cell was associated with improved prognosis. Therefore, the amounts of peripheral hematologic cells can reflect the inflammatory and immune changes.

Numerous studies showed that inflammatory-based prognostic indexes, such as PLR and NLR, were considered to have prognostic value for DLBCL. A retrospective study indicated that high PLR value was significantly related to poor prognosis to R-CHOP for DLBCL (27). Meanwhile, a meta-analysis revealed that high PLR was related to poor tumor behavior and poor OS with 1931 individuals, and the PLR might be an effective factor of poor prognosis for patients with DLBCL (28). A study of 505 patients proved that NLR may be a useful prognostic marker in OS and could predict the progression in patients with DLBCL (11). A recent meta-analysis revealed that the NLR was associated with worse OS and regarded as an inexpensive prognostic factor with 2297 patients with DLBCL (29). SII, based on neutrophil, platelet and lymphocyte, may reflect the inflammatory status and tumor activity more accurately than PLR and NLR, and SII has been proved to be an independent predictor in several malignancies, such as breast cancer and lung cancer (14, 15). However, data in non-Hodgkin’s lymphoma are limited and only one study has been performed to assess the impact of pretreatment SII in DLBCL. Yang et al. (30) enrolled 28 patients with testicular DLBCL and found that pretreatment SII was a negative prognostic factor for PFS. But it should be noted that the most common chemotherapy regimen was CHOP without rituximab and the population was small. Whether SII could influence the outcome of DLBCL patients is still unclear, so we conducted this retrospective study and hope that SII could be useful for predicting the prognosis in DLBCL.

In the present study, we reviewed 224 patients with DLBCL for their clinical and laboratory data and evaluated the prognostic impact of SII in DLBCL. We determined the cutoff value of 1046.1 for SII. Consistent with other solid cancers (16, 31), our results showed high SII was associated with more aggressive clinical features including high level of LDH, more advanced stage, poor PS, and high IPI score. Advanced disease, higher LDH and higher IPI score reflect a high tumor burden, leading to a negative prognosis (32). Neutrophils which exist in tumor microenvironment are capable of producing IL10 and CCL3 to promote tumor growth (19). Ji et al. (33) found that the serum CCL3 and IL-10 levels were significantly increased in DLBCL patients with high LDH levels. The above indicated that elevated neutrophils which mainly stimulated by cytokines produced by tumor cells in turn induced the release of chemokines and cytokines, thus promoted cancer growth. Meanwhile, The high SII at diagnosis negatively correlated with the overall response rate and the complete response rate significantly (p<0.001). These evidences prove that SII could reflect a higher tumor burden and predict chemosensitivity.

High SII had a significant negative impact on the 3-year PFS and the OS as compared with low SII. Other inflammation factors including PLR and NLR also showed the similar result. In univariate analysis, the NLR, PLR and SII could be used to judge the prognosis of patients. However, according to the multivariate analysis, only the SII was a prognostic factor for patients with DLBCL. The ROC curve analysis showed that the SII was more accurate and effective in predicting the outcomes of patients when compared with NLR or PLR.

Due to the high prevalence of hepatitis B infection in the Chinese population, we studied the prognosis of patients with HBsAg-positive. We found that high SII, high IPI score, HBsAg-positive and older age were significant prognostic factors for OS in patients with DLBCL. Based on this finding, a new nomogram included high SII, high IPI score, HBsAg-positive and older age was established to predict the prognosis of patients. The ability of the nomogram to predict the outcome was more accurate than that of IPI(c index 0.791 *vs*. 0.716). This result suggested that SII, when combined with IPI, age and HBsAg status, could provide more accurate prognostic information in patients with DLBCL, identify high-risk patients early and provide support for selecting individualized treatment strategy.

Nonsteroidal anti-inflammatory drugs (NSAIDs) could inhibit the enzyme cyclooxygenase (COX) to reduce inflammation (34). Additionally, aspirin, a kind of NSAIDs, have been found to induce apoptosis and inhibit tumor invasion by decreasing the expression of B-cell lymphoma 2 (BCL-2) and inhibiting NFκB signaling pathway (35). Previous studies have reported that NSAIDs is associated with reduced risk of cancer-related death by inhibit tumor growth in several malignancies (36, 37). Further research is necessary to investigate the therapeutic effect of NSAIDs in improving prognosis in patients with DLBCL.

There were some limitations in this study. First, it was a single-center and retrospective study and the number of patients was not large enough. Therefore, there were some selection and analytical biases. Second, there were no consistent cutoff values for SII in different studies, which led to the difficulty of comparing our results with other studies. Third, this study did not evaluate the association between prognosis and the changes of SII before and after treatment, which could reflect the change of inflammatory status to predict the survival and recurrence. Fourth, we did not investigate the link between SII and recurrent mutations reported in DLBCL.

In conclusion, this is the first analysis to demonstrate that the high SII as a reproducible and easily assessable prognostic biomarker is able to predict poor outcome in DLBCL patients. Maybe SII can be combined with other biomarkers to identify high risk patients and instruct treatment. But larger prospective clinical trial is warranted to validate this result and explore the mechanistic explanation for association between the SII and clinical outcome in DLBCL patients.

## Data Availability Statement

The raw data supporting the conclusions of this article will be made available by the authors, without undue reservation.

## Ethics Statement

The studies involving human participants were reviewed and approved by Ethics Committee of The Second Affiliated Hospital of Zhejiang University School of Medicine. The patients/participants provided their written informed consent to participate in this study.

## Author Contributions

ZW designed the study, performed the data analysis, and drafted the manuscript. JZ performed the data analysis and drafted the manuscript. SL participated in the data acquisition and drafted the manuscript. XZ designed the study and revised the manuscript. All authors contributed to the article and approved the submitted version.

## Conflict of Interest

The authors declare that the research was conducted in the absence of any commercial or financial relationships that could be construed as a potential conflict of interest.
